# ATP modulates SLC7A5 (LAT1) synergistically with cholesterol

**DOI:** 10.1038/s41598-020-73757-y

**Published:** 2020-10-07

**Authors:** Jessica Cosco, Mariafrancesca Scalise, Claire Colas, Michele Galluccio, Riccardo Martini, Filomena Rovella, Tiziano Mazza, Gerhard F. Ecker, Cesare Indiveri

**Affiliations:** 1 Department of DiBEST (Biologia, Ecologia, Scienze Della Terra) Unit of Biochemistry and Molecular Biotechnology, University of Calabria, via Bucci 4C, 87036 Arcavacata di Rende, Italy; 2 Department of Pharmaceutical Chemistry, University of Vienna, Althanstrasse 14, 1090 Wien, Austria; 3 CNR Institute of Biomembranes, Bioenergetics and Molecular Biotechnologies (IBIOM), via Amendola 122/O, 70126 Bari, Italy

**Keywords:** Proteins, Biochemistry, Molecular modelling

## Abstract

The plasma membrane transporter hLAT1 is responsible for providing cells with essential amino acids. hLAT1 is over-expressed in virtually all human cancers making the protein a hot-spot in the fields of cancer and pharmacology research. However, regulatory aspects of hLAT1 biology are still poorly understood. A remarkable stimulation of transport activity was observed in the presence of physiological levels of cholesterol together with a selective increase of the affinity for the substrate on the internal site, suggesting a stabilization of the inward open conformation of hLAT1. A synergistic effect by ATP was also observed only in the presence of cholesterol. The same phenomenon was detected with the native protein. Altogether, the biochemical assays suggested that cholesterol and ATP binding sites are close to each other. The computational analysis identified two neighboring regions, one hydrophobic and one hydrophilic, to which cholesterol and ATP were docked, respectively. The computational data predicted interaction of the ϒ-phosphate of ATP with Lys 204, which was confirmed by site-directed mutagenesis. The hLAT1-K204Q mutant showed an impaired function and response to ATP. Interestingly, this residue is conserved in several members of the SLC7 family.

## Introduction

The human LAT1 transporter (SLC7A5) is one of the seven members of the SLC7 family characterized by the association with ancillary glycoproteins belonging to the SLC3 family, namely SLC3A1 and SLC3A2^1,2^. The formation of these heterodimers represents a peculiar feature for mammalian transporters in terms of both structural and functional properties. The heterodimer is formed via a disulfide (S–S) between a conserved Cys residue of the SLC7 member(s), representing the light subunit, and a conserved Cys residue of the SLC3 member(s), representing the heavy subunit. The actual biological significance of such an interaction is not completely defined neither in terms of evolution nor in terms of regulatory aspects even though it is plausible that the ancillary glycoproteins serve as a chaperone for the light subunit to reach the definitive location in the cell membrane^1,2^. In particular, LAT1 forms a functional heterodimer with SLC3A2, commonly known as CD98 or 4F2hc. Over the years, it has been demonstrated, both in intact cells (ex vivo) and in proteoliposomes (in vitro), that the sole competent subunit for the transport function is LAT1, while CD98 does not play any role for the intrinsic transport function^3^. In good agreement with this observation, CD98 is a multifunctional protein that plays biological roles independently from the interaction with membrane transporters indicating that this protein is not a “simple” chaperone^4^. The functional properties of LAT1 have been investigated for decades and upon the description of its substrate specificity, it is now well assessed that the protein is involved in the distribution of essential amino acids in cells by catalyzing an amino acid antiport. Physiologically, histidine is the substrate preferentially exported from the cell interior to allow the other essential amino acids to be absorbed^3,5^. LAT1 has a quite narrow tissue distribution: it is mainly expressed in placenta and the blood–brain barrier. In these districts, the supply of essential amino acids is fundamental for normal cell growth and development^2^. Indeed, mice embryos KO for LAT1 are not vital, indicating that the absence of this protein is life-threatening or, even, not compatible with life^6^. Besides this experimental observation, the relevance of hLAT1 for the human being is testified by the virtual absence of diseases so far known, characterized by the complete lack of protein expression. In good agreement with this postulate, it has been recently demonstrated, by experiments conducted on mice and in vitro models, that two natural point mutations of LAT1 are responsible for the appearance of a familiar form of autism spectrum disorders (ASD) characterized by a lower supply of essential amino acids in brain^5^. On the other way round, the over-expression of LAT1 is now considered a hallmark of several human cancers even if they originate from tissues that normally do not express LAT1^2,7^. Notwithstanding the high frequency of this phenomenon, its biological significance is not yet completely defined. Over the years, it has been proposed that cancer cells over-express LAT1 for absorbing glutamine and leucine, through a cycle with the glutamine transporter ASCT2, to supply the high demand of these amino acids^8^. Later on, it has been demonstrated that glutamine is not a good substrate of LAT1, in both directions of transport cycle^3^. Therefore, the high expression of LAT1 may be linked to the need for essential amino acids used in protein synthesis under the high proliferation conditions of cancer cells. However, LAT1 is not only required for sustaining cell divisions. It is not trivial that, among the transported substrates, there is likely one or more responsible for the additional role(s) in cancer cells. As an example, leucine is an allosteric regulator of the mitochondrial enzyme GDH that is responsible for the utilization of glutamine carbon skeleton in TCA, a feature typical of cancer cells^9,10^. Leucine is also one of the signals employed by cells to regulate their metabolism: in lysosomes, the master regulator of cell metabolism, mTORC1, senses leucine levels in both physiological and pathological conditions^11^. Furthermore, sestrin 2 has been recently described as the cytosolic sensor of leucine levels, linked to mTORC1^11^. Then, it is not a surprise that LAT1 is considered a hot protein and that over the years, efforts have been made to define structure/function relationships as well as regulatory properties with the main scope to specifically target this protein for pharmacological applications^12–14^. Interestingly, the 3D structure of the complex LAT1/CD98 has been recently solved opening important perspectives for structure/function relationship studies and drug design^15^. So far, only one compound which targets LAT1 reached the clinical trial for cancer treatment, i.e. the tyrosine analogue JPH203^16,17^. In the context of drug development, parallel to inhibitors, also prodrugs are designed given the ability of LAT1 to transport also thyroid hormones, L-DOPA and gabapentin, i.e. non amino-acid substrates^18^. The study of the regulators of LAT1 is, on the contrary, still *in nuce* and this represents a strong restrain for further understanding of LAT1 biology both in physiological and in pathological conditions. Therefore, in the present work, we aimed to investigate the modulation of LAT1 transport activity by physiological effectors, employing the experimental model of proteoliposomes. This tool gives the possibility of easily modifying the lipid composition of the membrane as well as precisely controlling the composition of the external and internal aqueous compartments of the protein-harboring vesicles^19^. We moved from the increasing evidence that cholesterol can regulate the features of several membrane transporters and that LAT1 shows potential binding sites for cholesterol^20^. Interestingly, we identified by in silico and in vitro studies a novel regulation by intracellular ATP which is synergistic with the effect of cholesterol. Moreover, the molecular basis of the pH sensitivity was also described, moving a step forward in completing the knowledge on human LAT1.

## Results

### Effect of cholesterol on the transport activity of hLAT1 in proteoliposomes

**Figure 1:**
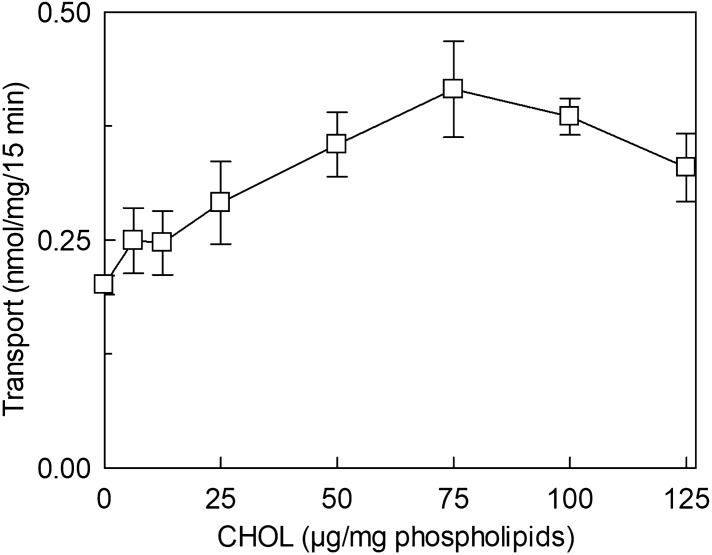
Effect of cholesterol on the transport activity of hLAT1. The purified protein was reconstituted in proteoliposomes as described in “Methods”. Transport assay was started adding 5 μM [^3^H]-histidine to proteoliposomes containing 10 mM histidine prepared with the indicated concentrations of cholesterol (CHOL). The transport was measured in 15 min according to the stop inhibitor method. Transport rate was expressed as nmol/mg in 15 min. Results are means ± SD of at least three independent experiments.

**Figure 2:**
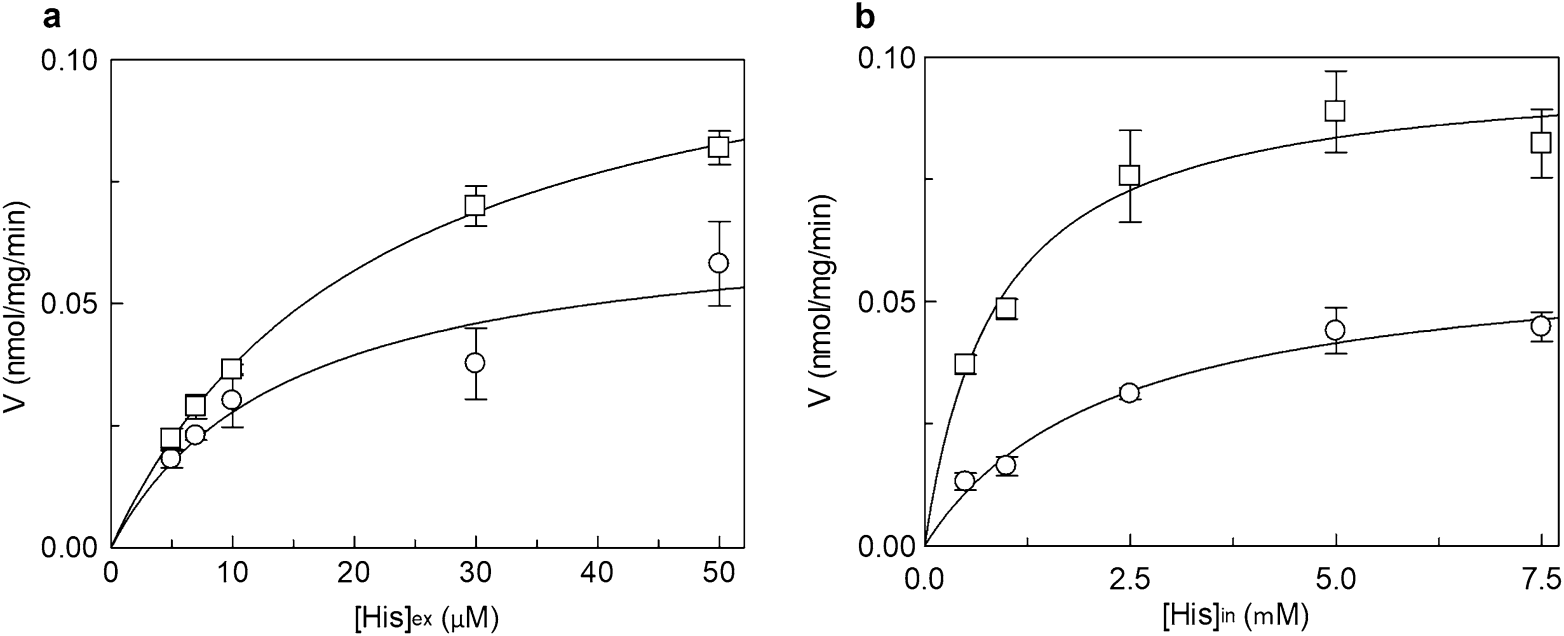
Effect of cholesterol on the kinetics of hLAT1. The purified protein was reconstituted using proteoliposomes prepared without (○) or with 75 μg cholesterol/mg phospholipids (□). The transport was measured in 15 min according to the stop inhibitor method. In (**a**), measurement of external Km. Transport rate was measured by adding [^3^H]-histidine at the indicated concentrations to proteoliposomes containing 10 mM histidine. Data were plotted according to the Michaelis–Menten equation as transport rate vs histidine concentration. In (**b**), measurement of internal Km. Transport rate was measured by adding 30 µM [^3^H]-histidine to proteoliposomes containing in the internal side indicated concentrations of histidine. Data were plotted according to the Michaelis–Menten equation as transport rate vs histidine concentration. Results are means ± SD of at least three independent experiments.

The effect of cholesterol (CHOL) on hLAT1 function was studied using the proteoliposome tool harboring the functionally active recombinant protein and assaying its function as [^3^H]-histidine_ex_/histidine_in_ antiport. The transport activity increased by increasing the cholesterol content in the proteoliposome lipids up to 75 μg cholesterol/mg phospholipids; at higher cholesterol concentration, the transport activity decreased (Fig. 1). To obtain information on the possible influence of cholesterol on the affinity for the substrate on the external or internal face of the protein, kinetic parameters were measured under the optimal condition of cholesterol concentration. It has to be stressed that the transporter is inserted in the proteoliposome membrane with the same orientation as in the cell membrane, therefore, the extraliposomal or the intraliposomal side corresponds to the extracellular or the intracellular side, respectively^3,21^. Figure 2a shows the transport rate dependence on external substrate concentration plotted according to Michaelis–Menten equation; a Km of 22.6 ± 4.3 µM (Fig. 2a, upper curve) was measured which was similar or only slightly higher than the external Km measured in the absence of cholesterol, that is 14.9 ± 2.7 µM (Fig. 2a, lower curve). This small variation correlates well with previous data obtained in cell systems in which the Km of the low-affinity substrate L-DOPA was shown to not be influenced by sequestration of cholesterol by methyl-cyclodextrin^20^. The Vmax of [^3^H]-histidine_ex_/histidine_in_ antiport increased in the presence of cholesterol (Fig. 2a) from 0.069 ± 0.0094 to 0.12 ± 0.012 nmol/mg/min, in line with the data of Fig. 1. Interestingly, when measuring the internal Km, a different behavior was observed with respect to the external one; indeed, the internal Km significantly decreased from 2.3 ± 0.6 mM in the absence of cholesterol to 0.87 ± 0.12 mM in the presence of cholesterol (Fig. 2b), indicating an increased internal affinity for the substrate. In good agreement with the data obtained when investigating the external side, the Vmax increased from 0.061 ± 0.0087 nmol/mg/min to 0.10 ± 0.0038 nmol/mg/min.

### Effect of nucleotides on the transport activity of hLAT1

**Figure 3:**
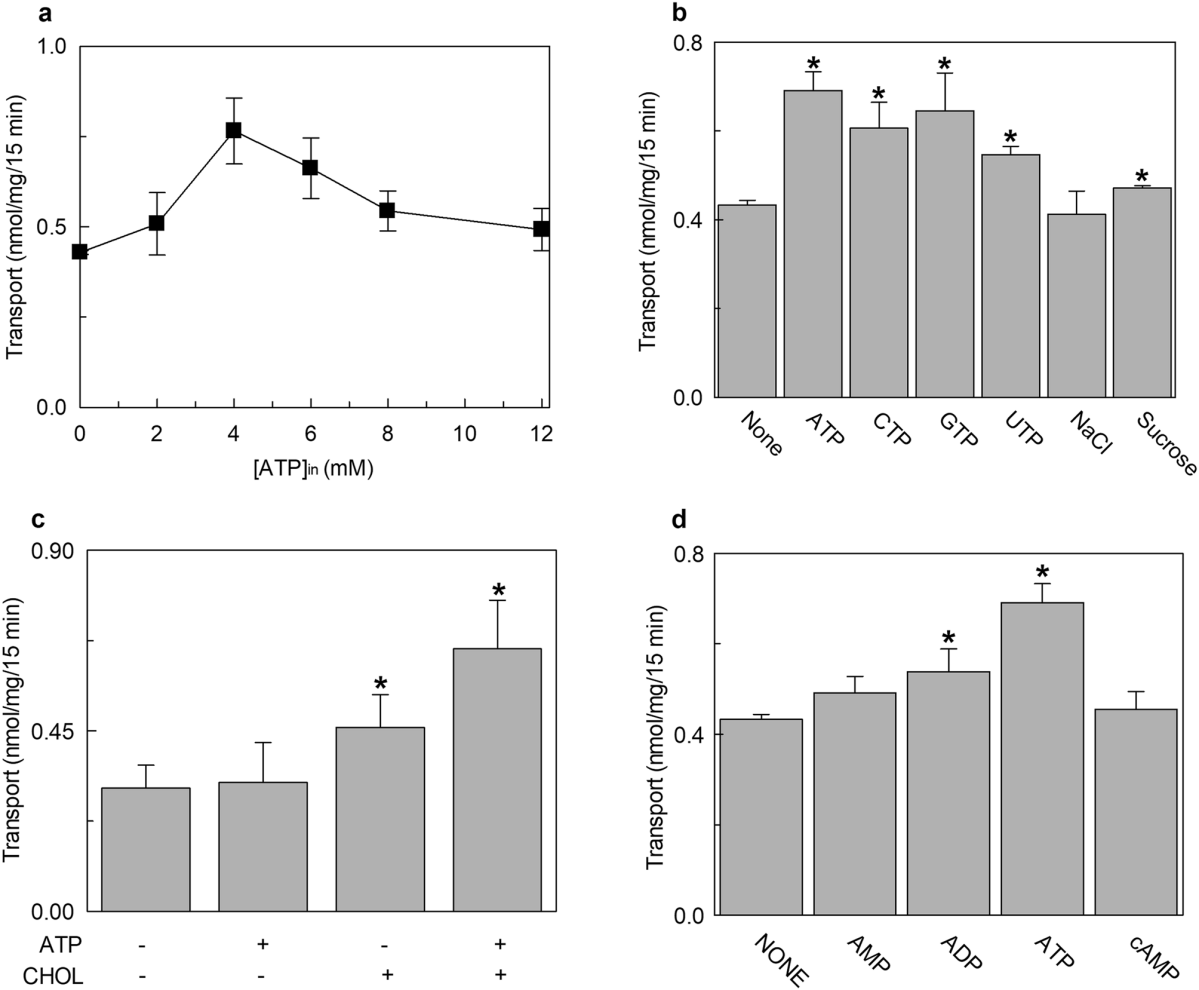
Effect of intraliposomal nucleotides on the transport activity of hLAT1. The purified protein was reconstituted in proteoliposomes prepared with 75 μg cholesterol/mg phospholipids as described in “Methods”. Transport assay was started adding 5 μM [^3^H]-histidine to proteoliposomes containing 10 mM histidine. The transport was measured in 15 min according to the stop inhibitor method. Transport rate was expressed as nmol/mg in 15 min. In (**a**), the dependence of hLAT1 transport activity on intraliposomal ATP. The indicated concentrations of ATP, buffered with 20 mM HepesTris pH 7.0, were added in the internal side of proteoliposomes. In (**b**), the effect of nucleotides on the transport activity of hLAT1. Intraliposomal compartment included 4 mM of the indicated nucleotides, buffered with 20 mM HepesTris pH 7.0. As an osmotic control, 12 mM NaCl or 12 mM sucrose was added in place of nucleotides. In (**c**), transport activity of the recombinant hLAT1. Proteoliposomes were prepared without (−) or with (+) 75 μg cholesterol/mg phospholipids as indicated; 4 mM ATP, buffered with 20 mM HepesTris pH 7.0, was included (+) or not (−) in the intraliposomal compartment as indicated. (**d**) Effect of AMP, ADP and cAMP on the transport activity of hLAT1. Proteoliposomes contained 4 mM of AMP, ADP, cAMP or ATP. Results are means ± SD of at least from three independent experiments. (*) Significantly different from the control (no addition in the intraliposomal compartment, none) as estimated by the Student's t-test (p < 0.05).

**Figure 4:**
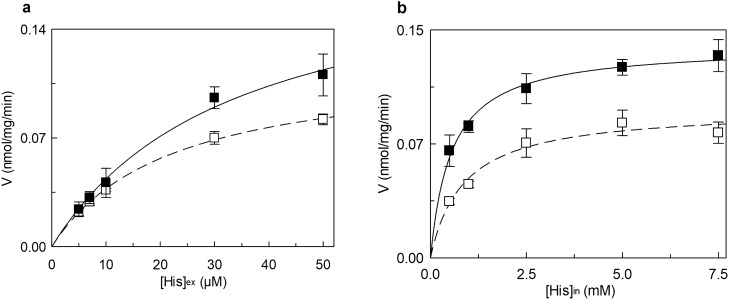
Effect of ATP on the kinetics of hLAT1. The purified protein was reconstituted in proteoliposomes prepared with 75 μg cholesterol/mg phospholipids as described in “Methods”, containing (■) or not (□) 4 mM ATP (in dotted line, data from corresponding Fig. 2). The transport was measured in 15 min according to the stop inhibitor method. In (**a**), measurement of the external Km. Transport rate was measured by adding [^3^H]-histidine at the indicated concentrations to proteoliposomes containing 10 mM histidine. Data were plotted according to the Michaelis–Menten equation as transport rate vs histidine concentration. In (**b**), measurement of the internal Km. Transport rate was measured by adding 30 µM [^3^H]-histidine to proteoliposomes containing the indicated concentration of histidine. Data were plotted according to the Michaelis–Menten equation as transport rate vs histidine concentration. Results are means ± S.D. of at least three independent experiments.

**Figure 5:**
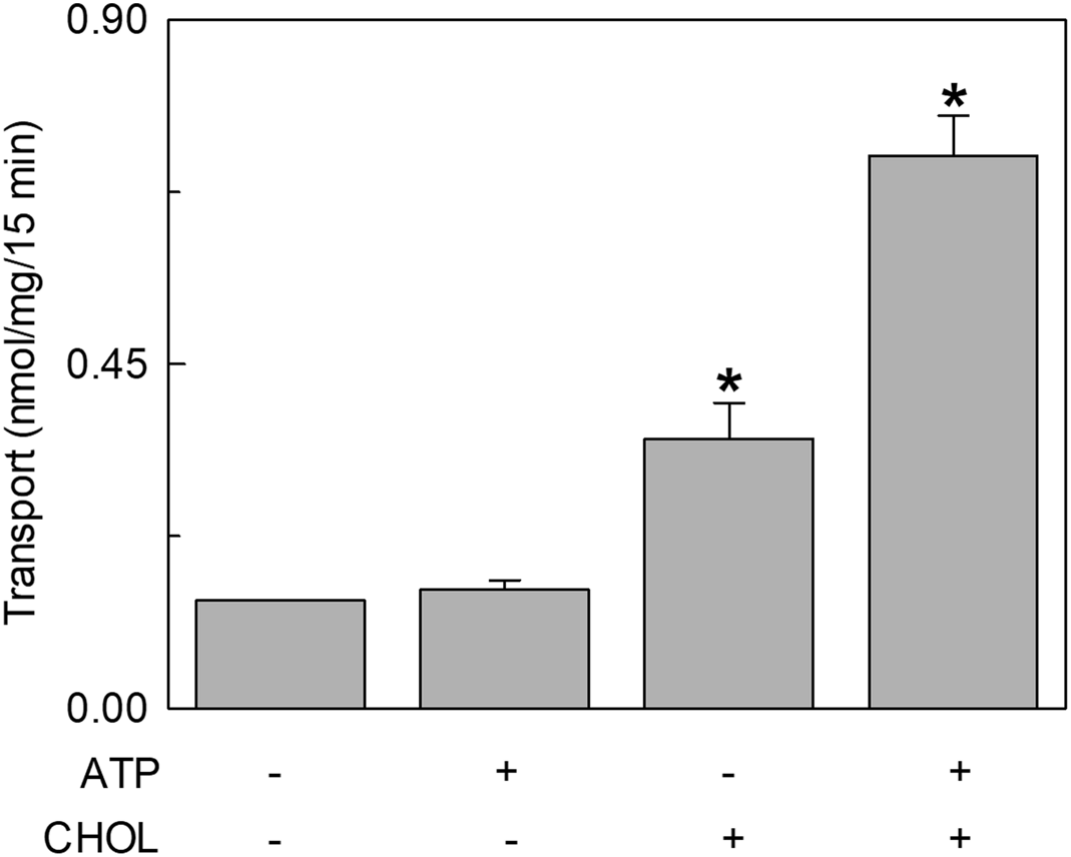
Effect of ATP on the transport activity of hLAT1 extracted from SiHa cells reconstituted in proteoliposomes. SiHa cell extract was prepared as described in “Methods”. Transport was started adding 5 μM [^3^H]-histidine to proteoliposomes containing 10 mM histidine prepared without (−) or with (+) 75 μg cholesterol/mg phospholipids as indicated; 4 mM ATP, buffered with 20 mM HepesTris pH 7.0, was included (+) or not (−) in the intraliposomal compartment as indicated. The transport was measured in 15 min according to the stop inhibitor method. Results are means ± S.D. of at least three independent experiments.

In the attempt of exploring possible cell regulators linked to the energy metabolism, nucleotides were tested since ATP and other nucleotides were previously found to influence the activity of transporters^22–25^. The [^3^H]-histidine_ex_/histidine_in_ antiport was measured in the presence of internal ATP under the optimal condition of cholesterol concentration. The transport activity increased by increasing the intraliposomal (intracellular) ATP concentration up to 4 mM and then decreased at higher concentrations (Fig. 3a). Then, other tri-phospho-nucleotides, namely CTP, UTP and GTP were tested in comparison with ATP. CTP, UTP and at a lower extent GTP, stimulated the transport function at a concentration of 4 mM (Fig. 3b). It has to be highlighted that 4 mM is in the range of the intracellular concentration of ATP but is much higher than the concentration of other nucleotides. Indeed, GTP, CTP and UTP did not exert any effect at concentrations from 0.1 to 0.3 mM (Supplementary Fig. 1a) which are closer to the physiological conditions^26–28^. To exclude the possibility that the activation by ATP was due to an osmotic effect or to the Na^+^ cation present with the commercial formulation of ATP, sucrose or NaCl were tested as a control. No, or very small effect was observed in either condition indicating that the effect of the nucleotide is specific (Fig. 3b). Very interestingly, the activation by ATP did not occur if cholesterol is omitted from the proteoliposome preparation. In fact, in the absence of cholesterol, the nucleotide exerted only a very small, if any, effect (Fig. 3c). A clear activation was indeed observed in the presence of 75 μg cholesterol/mg phospholipids, in agreement with the data of Fig. 3a. This result suggests that the sites of interaction of cholesterol and ATP may be close to each other. The data reported in Fig. 3b indicates that the base moiety is not crucial for the activation. To investigate if the effect could be influenced by the phosphate groups, the transport activity was measured in the presence of 4 mM AMP, ADP or cAMP in comparison to ATP. As shown in Fig. 3d, the extent of activation decreased by decreasing the number of phosphate groups. This indicates that three phosphates are necessary for the full effect. Interestingly, the effect exerted by ATP is in the same order of magnitude of that exerted by ATP-Magnesium (Supplementary Fig. 1b). The kinetic parameters were then evaluated in the presence of 4 mM ATP with respect to the control condition without ATP. As shown in Fig. 4a, in the presence of intraliposomal ATP the external Km was slightly increased (33.9 ± 4.4 μM) and the V_max_ was higher than the control, i.e. the condition without ATP. The internal Km was not significantly influenced by the presence of ATP (0.57 ± 0.14 mM), while the Vmax was increased (Fig. 4b). To exclude a possible influence of the ancillary protein CD98 on the effect of cholesterol and ATP on hLAT1, the protein extracted from SiHa cells that is in complex with CD98^3^ was employed. As shown in Fig. 5, the transport activity is influenced by cholesterol and/or ATP as the recombinant protein. Indeed, ATP stimulated the transport only in the presence of cholesterol while the nucleotide did not exert any effect in the absence of cholesterol.

The effect of ATP was also evaluated on the external side. The transport activity is 25% inhibited at 4 mM ATP (Supplementary Fig. 2). This concentration value is higher than the extracellular concentration of ATP^27,28^, therefore this inhibition cannot be considered physiologically relevant. Moreover, this data confirms that the transporter is asymmetrical and inserted into the proteoliposome membrane with a right-side-orientation with respect to the native membrane as previously described^21^.

### Computational analysis of cholesterol and ATP interaction with hLAT1

**Figure 6:**
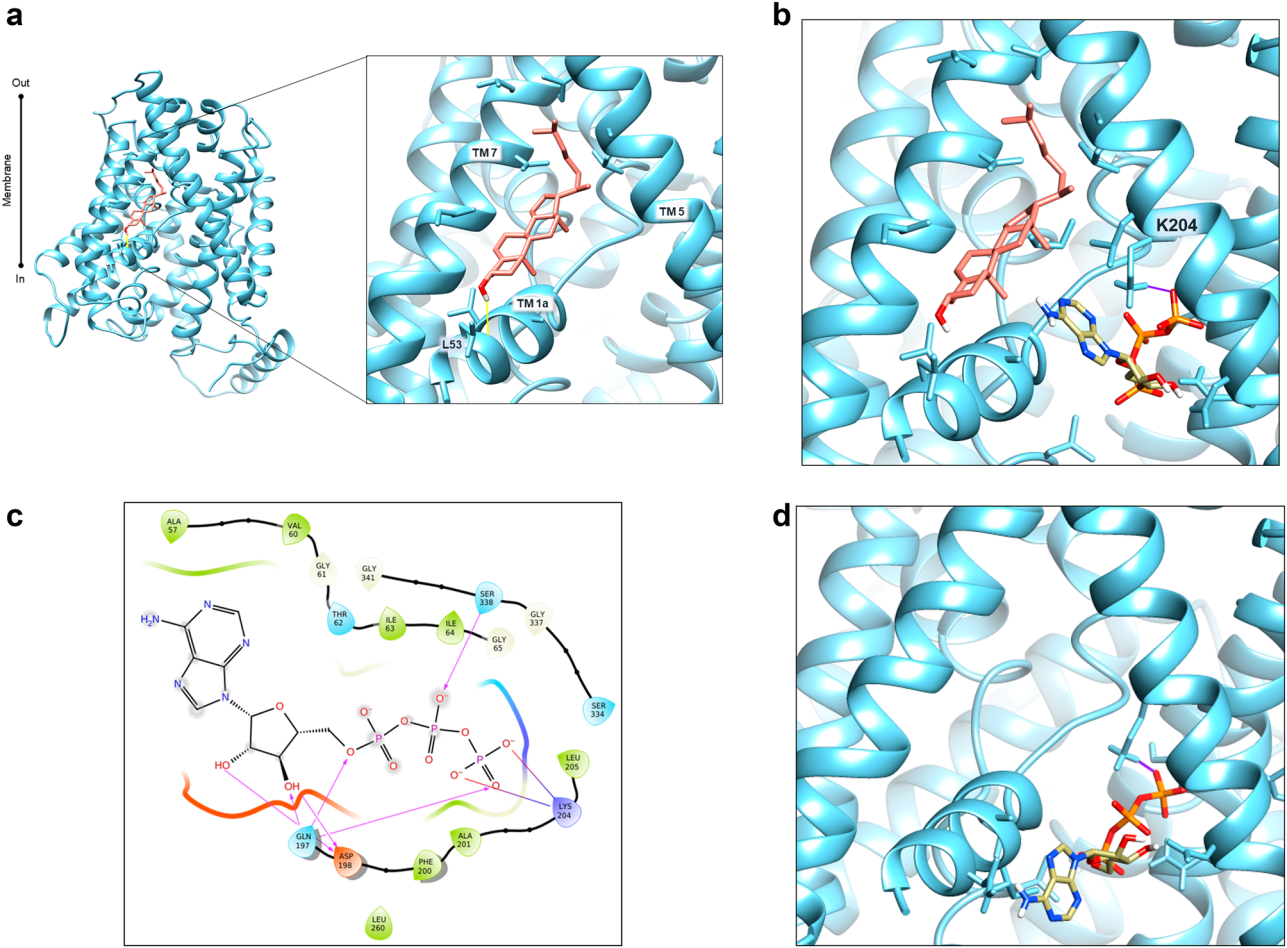
Docking analysis of hLAT1. Docking analysis in site 1 of hLAT1. The cryo-EM structure of hLAT1 in inward conformation (PDB ID: 6IRT, chain B) was represented as ribbon (sky blue) using Chimera v.1.7 software (https://www.cgl.ucsf.edu/chimera). Docking analysis was performed using InducedFit docking from Schrödinger-Maestro v11.3^65^ as described in “Methods”. In (**a**), molecular docking of cholesterol (in salmon) in the site 1 (TM 1a, TM5 and TM7). Residues belonging to cholesterol site are represented as sticks. This pose has a docking score of -5.54 and a MMGBSA binding energy of − 46.66 kcal/mol. The membrane and intracellular/extracellular environment are indicated. In (**b**), molecular docking of ATP in site 1 in the presence of cholesterol. The best pose from cholesterol docking was used to dock ATP. Lys 204 represented in stick makes an electrostatic bond (in violet) with the γ-phosphate of ATP. This pose has a docking score of − 6.92 and a MMGBSA binding energy of − 8.97 kcal/mol. In (**c**), 2D visualization of hLAT1 interaction with ATP. The arrows indicated the residues involved in the binding of ATP. In (**d**), molecular docking of ATP in site 1 in the absence of cholesterol; in violet, electrostatic bond with Lys 204 represented in stick. The pose has a docking score of − 6.336.

To investigate the interaction of cholesterol and ATP with hLAT1 on a molecular level, computational analysis was performed (Supplementary Fig. 3). The structure of the hLAT1 (PDB ID: 6IRT, chain B)^15^ has been used for the analysis. The initial step consisted in identifying hydrophobic and hydrophilic regions on the surface of hLAT1 using SiteMap^29,30^. Ten sites have been predicted with a score from 0.888 to 1.015, i.e. the optimal range for a reliable SiteMap prediction (Supplementary Fig. 3)^29^. Interestingly, site **d** corresponded to one of the lipid-like densities observed in the cryo-EM structure of hLAT1 (Supplementary Fig. 4). In this site, the very conserved **L**L**Y**AFS**K** motif is present (Supplementary Fig. 4), well-acknowledged as a cholesterol binding motif CRAC^31^. However, our attention for deepening the analysis of cholesterol and ATP binding sites was focused on site **h,** due to the presence of a hydrophilic surface close to a hydrophobic one (Supplementary Fig. 3). It is worth to note that site **h** includes two hydrophobic subsites, namely subsite 1 and subsite 2. Only subsite 1 is close to a hydrophilic region (identified in red/blue surfaces by SiteMap—Supplementary Fig. 3) thus being in line with our working hypothesis, based on biochemical data, that the binding sites for cholesterol and ATP should be close to each other. Moreover, subsite 1 includes also the residues previously predicted to be involved in cholesterol binding by sequence alignments with the *Drosophila melanogaster* dopamine transporter dDAT, sharing a similar fold and also presenting a cholesterol modulated activity^20^. Subsequently, docking of cholesterol was performed after generating a grid on the subsite 1 of site **h**. To obtain a more reliable result, an Induced Fit docking was performed as it incorporates side chains mobility of the binding site. After clustering of all poses retrieved, the pose with the lowest docking score (− 5.54) was selected from the most populated cluster (Fig. 6a). In this site cholesterol is accommodated by hydrophobic residues of the helices 1a, 5 and 7 such as L53, V56, A57, L208, A209, I212, I284, V288, L291. As described above, subsite 1 has in its vicinity a hydrophilic pocket that might accommodate ATP (Fig. 6a). Thus, Induced Fit docking of ATP was performed on this region (Fig. 6b). The ϒ-phosphate has an electrostatic interaction with the Lys 204. The ribose group was predicted to interact with the residues Gln 197 and Asp 198. Besides the interaction with the ribose group, Gln 197 interacts also with the α-phosphate. The β-phosphate forms a hydrogen bond with Ser 338 and (Fig. 6c). Interestingly, in the absence of cholesterol, the docking of ATP was different with the nucleotide remaining in a more external position (Fig. 6d) indicating that its binding is influenced by the presence of cholesterol in the subsite 1. Next, we conducted a blind docking of ATP in the presence of cholesterol in subsite 1 using AutoDock Vina^32^, which resulted in a single cluster located in the region of the hydrophilic surface of pose **h** (Supplementary Fig. 5). These results gave us further confidence in our ATP binding mode prediction.

### Basic functional characterization of site-directed mutant hLAT1-K204Q

**Figure 7:**
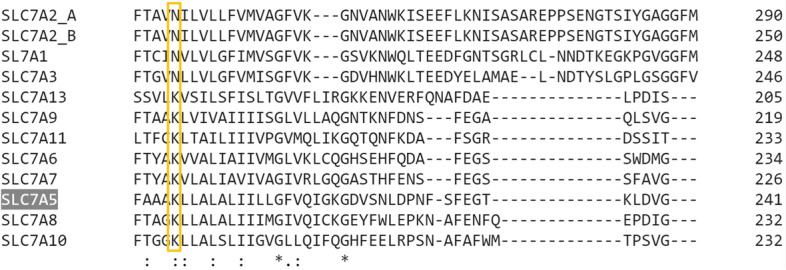
Multiple alignments of SLC7 protein sequences. Indicated SLC7 members were aligned using the software Clustal Omega as described in “Methods” (https://www.ebi.ac.uk/Tools/msa/clustalo)*.* In the yellow box, the conservation of Lys 204 (yellow box) of SLC7A5 is shown in comparison to the other members of the SLC7 family.

**Figure 8:**
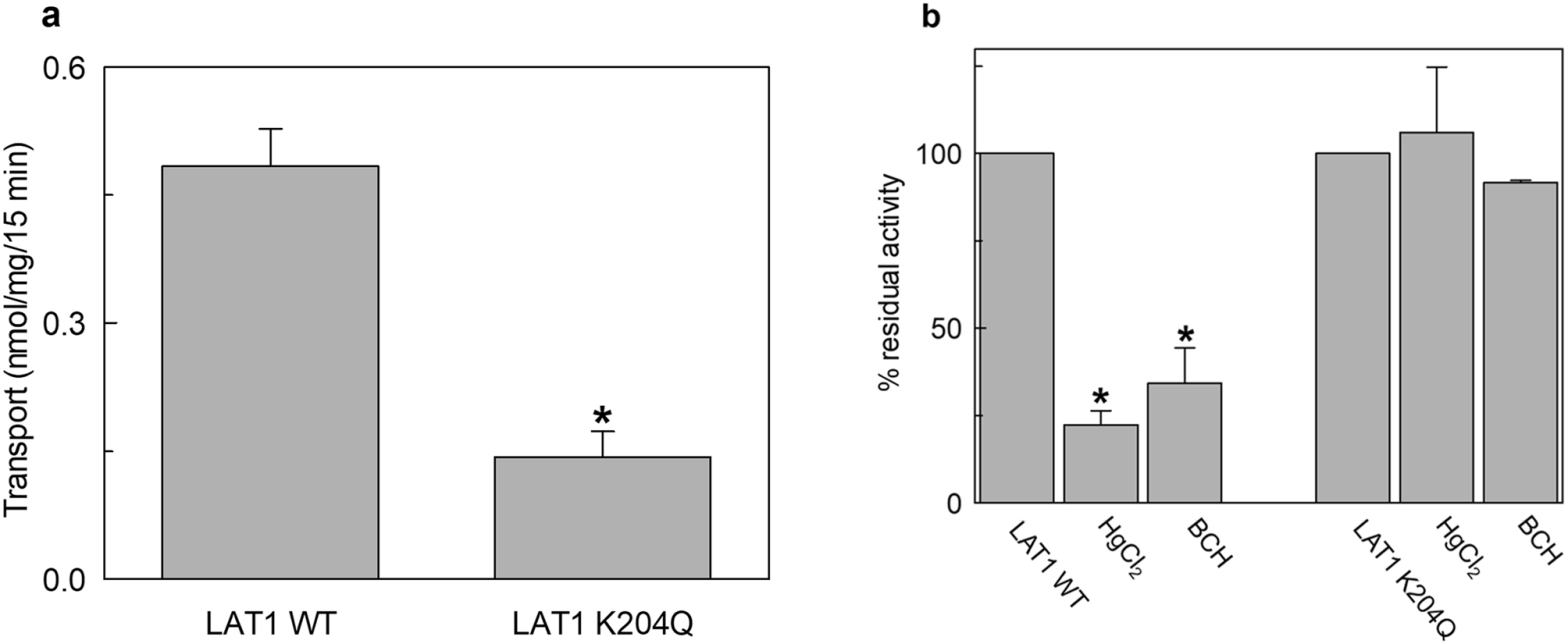
Transport activity of the recombinant hLAT1 WT or hLAT1-K204Q mutant. The mutant protein was prepared as described in “Methods”. The purified hLAT1-WT or hLAT1-K204Q proteins were reconstituted in proteoliposomes prepared with 75 μg cholesterol/mg phospholipids as described in “Methods”. Transport assay was started by adding 5 μM [^3^H]-histidine to proteoliposomes containing 10 mM histidine. The transport was measured in 15 min according to the stop inhibitor method. In (**a**), comparison of the transport activity of hLAT1-WT or hLAT1-K204Q mutant. Transport rate was expressed as nmol/mg in 15 min. In (**b**), the effect of HgCl_2_ and BCH on transport activity of hLAT1-WT or hLAT1-K204Q mutant. During transport assay, 15 μM HgCl_2_ and 5 mM BCH were added in the extraliposomal side together with the radiolabeled substrate. Transport was indicated as % residual activity with respect to the control (i.e. proteoliposomes with no externally added inhibitor). Results are means ± SD of at least three independent experiments. (*) Significantly different from the control (no externally added inhibitor) as estimated by the Student's t-test (p < 0.05).

**Figure 9:**
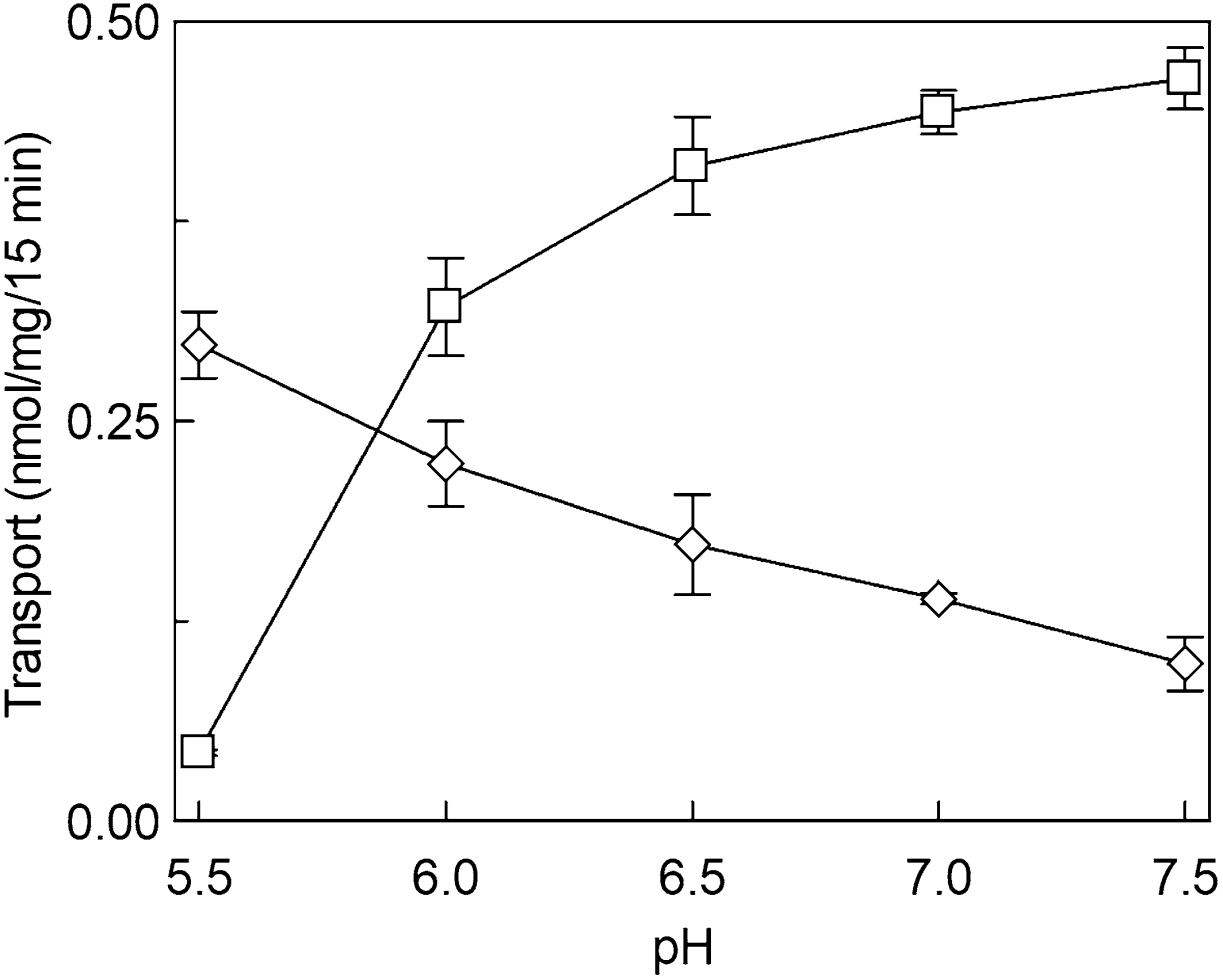
Effect of pH on the transport activity of hLAT1-WT or hLAT1-K204Q mutant. The purified proteins, WT (□) and K204Q (◊), were reconstituted in proteoliposomes prepared with 75 μg cholesterol/mg phospholipids as described in “Methods” and buffered with 20 mM Hepes Tris at the indicated pH. Transport was started by adding 5 μM [^3^H]-histidine to proteoliposomes containing 10 mM histidine. The transport was measured in 15 min according to the stop inhibitor method. Transport rate was expressed as nmol/mg in 15 min. The pH was equal in both the internal and external sides of proteoliposomes. Results are means ± SD of at least three independent experiments.

**Figure 10:**
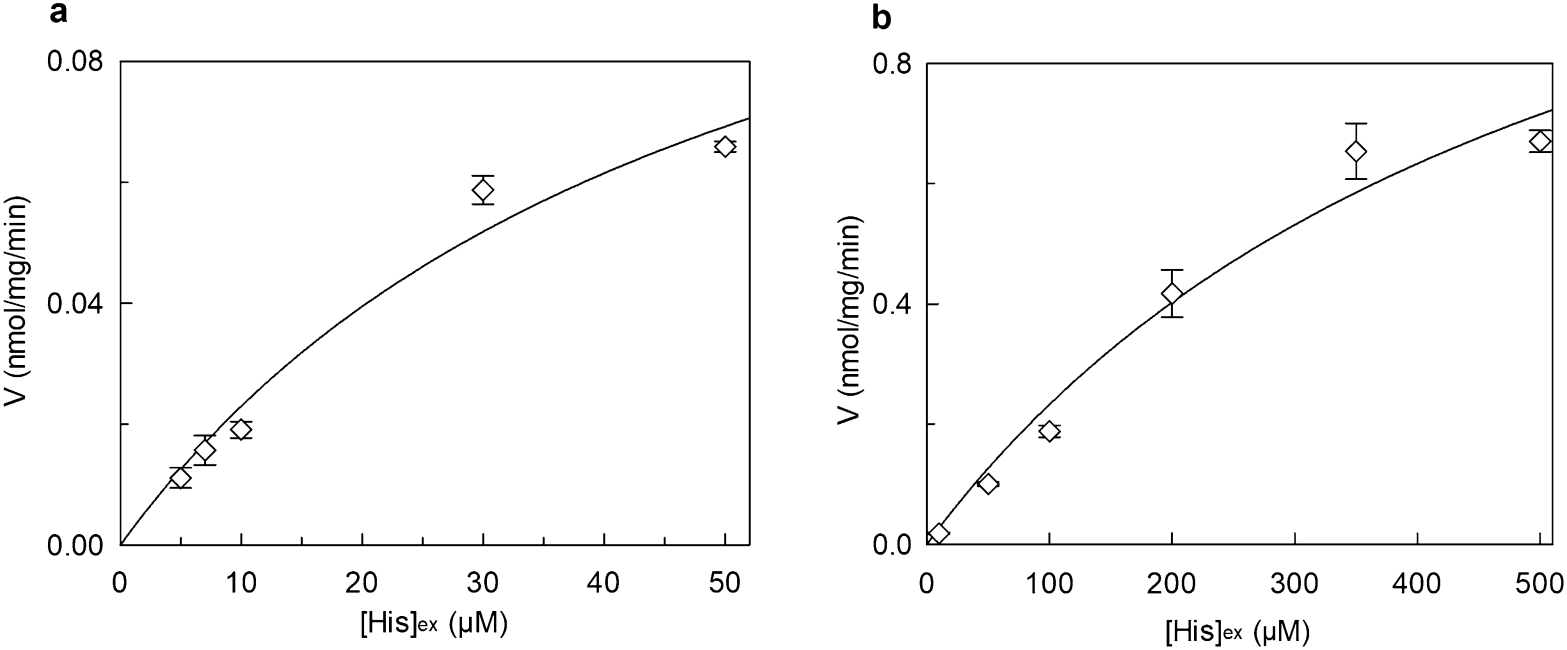
Effect of pH on external Km of hLAT1-K204Q mutant. The purified protein was reconstituted using proteoliposomes prepared with 75 μg cholesterol/mg phospholipids. The transport was measured in 15 min according to the stop inhibitor method. Transport rate was measured by adding [^3^H]-histidine at the indicated concentrations to proteoliposomes containing 10 mM histidine. Data were plotted according to the Michaelis–Menten equation as transport rate vs histidine concentration. In (**a**), proteoliposomes were prepared at pH 6.0 and substrate was buffered with 20 mM HepesTris pH 6.0. In (**b**), proteoliposomes were prepared at pH 7.0 and substrate was buffered with 20 mM HepesTris pH 7.0. Results are means ± SD of at least three independent experiments.

The docking analysis together with the biochemical data suggested focusing on Lys 204 since this residue establishes an electrostatic interaction with ϒ-phosphate, crucial for the ATP effect (Figs. 3d and 6c). Moreover, Lys 204 is conserved in the SLC7 family members whose transport function is already characterized (Fig. 7). Therefore, Lys was mutated to Gln to retain the hydrophilic nature and the size of the residue, while removing the positive charge. Despite the conserved features, the hLAT1-K204Q at pH 7.0 only exhibited 15% activity when compared to the WT (Fig. 8a). Another peculiar feature of this mutant was the loss of sensitivity towards inhibitors both competitive and covalent ones (Fig. 8b), indicating that the substrate-binding site of hLAT1 might be compromised by the mutation. The importance of Lys 204 for basic transport function of hLAT1 allowed us to hypothesize that its positive charge could be involved in the pH sensitivity of the protein. In contrast with previous findings^1,18,33^, we observed that the transport activity of hLAT1 is pH-dependent, being maximal at neutral pH but much lower at acidic pH (Fig. 9). Surprisingly, the hLAT1-K204Q exhibited an inverted pH dependence with the lowest activity at pH 7.5, while the activity at pH 6.0 was comparable to that of the WT at pH 7.0 (Fig. 9). Kinetics of transport was also studied and Km towards histidine was measured at both pH 7.0 and pH 6.0. The Km values were 549 ± 108 µM or 51.4 ± 7.5 µM at pH 7.0 (Fig. 10a) or pH 6.0 (Fig. 10b), respectively. In line with the data of Fig. 9, the V_max_ value at pH 6.0 is one order of magnitude higher than that at pH 7.0. Both the values were higher than that of the WT protein confirming the role of Lys 204 in the transport process and in substrate recognition (Fig. 8). This is in agreement with the location of this Lys residue in the proximity of the substrate-binding site (Supplementary Fig. 6).

### Effect of the K204Q mutation on ATP and cholesterol dependence

**Figure 11:**
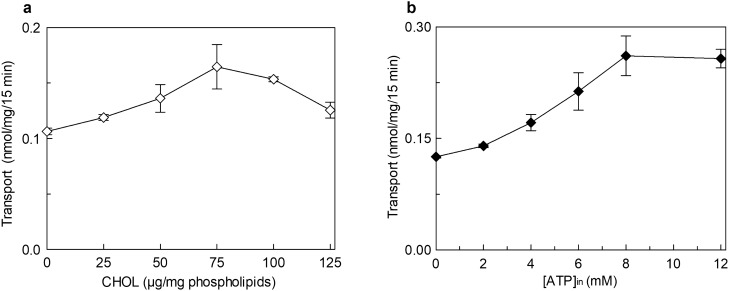
Effect of cholesterol and ATP on the hLAT1-K204Q mutant. The purified protein was reconstituted as described in “Methods”. Transport was started adding 5 μM [^3^H]-histidine to proteoliposomes containing 10 mM histidine. The transport was measured in 15 min according to the stop inhibitor method. Transport rate was expressed as nmol/mg in 15 min. In (**a**), the effect of cholesterol on the transport activity of hLAT1-K204Q mutant. Proteoliposomes were prepared with indicated concentrations of cholesterol (CHOL). In (**b**), the effect of internal ATP on transport activity of hLAT1-K204Q mutant. The indicated concentrations of ATP, buffered with 20 mM HepesTris pH 7.0, were added in the internal side of proteoliposomes prepared with 75 μg cholesterol/mg phospholipids. Results are means ± SD of at least three independent experiments.

After the basic functional characterization of the mutant, we investigated the dependence on cholesterol and ATP (Fig. 11). Interestingly, the mutant is activated by cholesterol similarly to the WT protein (Fig. 11a). In contrast, regarding the ATP dependence, the hLAT1-K204Q showed different behavior in comparison to the WT protein. As reported in Fig. 11b, the hLAT1-K204Q showed a shift in the ATP concentration required for transport stimulation. Noteworthy, the activity of the mutant was only slightly stimulated at 4 mM but reached the maximum of activation at 8 mM ATP (Fig. 11b). These results, which are quite different from those of the hLAT1 WT, indicated that the residue Lys 204 is crucial for ATP sensitivity in hLAT1.

## Discussion

The role of cholesterol in modulating the transport activity of hLAT1 is described in this work. The interaction of cholesterol with hLAT1 was previously hypothesized based on the identification of conserved motifs within proteins structurally related to hLAT1^20^ such as dDAT^34^ and hSERT^35^. Then, the recently solved 3D structure of hLAT1 in complex with hCD98 showed some lipid sites probably occupied by cholesteryl hemisuccinate added to the protein during purification^15^. The same authors showed that the addition of cholesterol to proteoliposomes harboring the LAT1-CD98 complex led to stimulation of leucine uptake. Transport modulation by cholesterol revealed to be a particularly interesting issue, since other transporters belonging to different SLC families were found to be modulated by this lipid as well^20,34,36–42^. Therefore, we deepened this aspect by using biochemical approaches together with computational analysis. From the collected data, it emerged that cholesterol added to the proteoliposomes indeed stimulated the transport activity measured as antiport of the high-affinity substrate histidine (Fig. 1). The intrinsic feature of proteoliposome tool allowed us to investigate in further detail the effect of cholesterol on hLAT1 function in terms of kinetic parameters. The affinity for the substrate on the external face of the transporter was not, or only slightly, modified by cholesterol (Fig. 2a). These findings correlate well with previous results on hLAT1, obtained by a different experimental approach consisting in sequestering cholesterol from the cell membrane by methyl-cyclodextrin^20^. Moreover, the internal affinity for the substrate, which cannot be approached using the intact cell system, was also investigated. Differently from the external side, the internal affinity was strongly increased suggesting a stabilization of the hLAT1 inward open conformation by cholesterol (Fig. 2b). This differs from data on hSERT, obtained by studies performed with inhibitors, showing that cholesterol favours the outwardly open conformation^35^. As in the case of other membrane proteins^22,37–44^, cholesterol effects might be not exclusively due to an action on the membrane fluidity/arrangement, but they could be related to a physical interaction with the protein. Very probably, cholesterol binds to hLAT1 in more than one site as suggested by the patch of densities reported in the 3D structure, and in agreement with our computational analysis (Supplementary Fig. 3). At this stage is not possible to evaluate the exact contribution of each cholesterol binding site to the transport activity of LAT1. Interestingly, some of the identified sites, namely **d**, **e**, **f**, **g** and **h** are coincident with those previously predicted^15,20,45^ (Supplementary Fig. 3). It is important to highlight that cholesterol can bind to membrane proteins in annular or non-annular sites, engaging distinct types of interaction: (1) annular sites are less specific and devoted to mediate the interaction of a hydrophobic transmembrane segment with the phospholipid bilayer; (2) non-annular sites engage larger protein domains and are involved in the regulation of the protein function^31^. Therefore, it is more likely that the effect on substrate affinity could be due to the interaction of cholesterol with sites showing non-annular features. The selection of the most plausible cholesterol-binding site was supported by the new collateral finding of regulation of transport activity by intraliposomal ATP (Fig. 3). Indeed, the stimulatory effect of ATP was only exerted in the presence of cholesterol, thus strengthening the hypothesis that the respective binding sites are close to each other. The extent of stimulation by cholesterol is similar to that previously described for the same^15,20^ or other transporters^37–39,41^. The synergistic effect by cholesterol and ATP reaches a factor higher than 3. In line with a physiological role of such a modulation, ATP-Magnesium is able to exert the same activation of free ATP. This indicates that the LAT1 bound form is ATP, as observed in other cases^46,47^. It has to be stressed that this phenomenon was observed also on the native transporter. This is in line with our previous findings demonstrating that all the transport features are virtually identical in the native and the recombinant hLAT1^3^. Very interestingly, the docking analysis of ATP on the whole hLAT1 structure resulted in a single cluster of interactions coincident with the hydrophilic pocket close to subsite 1 in site **h** (Fig. 6a and Supplementary Fig. 5). Altogether, the described observations indicated that binding of ATP in subsite 1 (of site **h**) might explain the synergistic effect of ATP and cholesterol. It has to be noted that ATP has a secondary role in the transport modulation with respect to cholesterol, since it does not influence substrate affinity, but only the transport rate (Fig. 4). This phenomenon may be interpreted also in terms of docking analysis of ATP performed on the homology model of hLAT1 in an outward open conformation (Supplementary Fig. 7). In fact, the hydroxyl moiety of cholesterol establishes hydrogen bond interactions alternatively with the backbone atoms of Leu 53 (Fig. 6a) or the side chain of Asp 198 (Supplementary Fig. 7) in the inward or outward conformation, respectively. In the outward state, Lys 204 that is the major determinant for ATP coordination is buried. This let us hypothesize that the binding of ATP only occurs in the inward open conformation in line with its accessory role with respect to cholesterol.

As a serendipity event, the residue underlying the ATP effect gave further insights into the hLAT1 structure/function relationships. The homologous residue in bacterial ApcTs has a role in conformational changes and pH response^48–50^. Thus, we investigated the relationships of Lys 204 with the pH response of hLAT1. First, we revealed that, contrarily to previous assumptions^1,18,33^, the activity of hLAT1-WT is pH-dependent (Fig. 9). This novel finding may be due to the better suitability of the in vitro system of proteoliposomes for investigating the pH dependence^19^. Interestingly, the loss of the positive charge of Lys 204 in the hLAT1-K204Q triggered an inverted pH dependence with respect to the hLAT1-WT. Moreover, as previously supposed for the bacterial homologue GkApcT^50^, Lys 204 has also a crucial role in the layout of the active site: the hLAT1-K204Q shows an impairment of the affinity towards histidine and loses the response to inhibitors targeting the substrate-binding site (Fig. 8). Indeed, Lys 204 is not far from residues involved in the substrate binding and translocation^15,21^ (Supplementary Fig. 6). The influence of Lys 204 on the active site may be mediated by the residues present in the kink of helix 1 interposed between the active site and Lys 204 (Supplementary Fig. 6). This correlates with the finding that this helical domain most probably moves during the conformational changes necessary for translocation as also recently proposed^45,51^. The stimulation by ATP may have a biological significance linked to the metabolic state of the cells. It is well known that ATP acts also as a metabolic regulator on a great number of enzymes and, in some cases, of transporters^22–25,42^. To our knowledge, this is the first case of a transporter of the SLC7 family, showing a regulation by ATP. Therefore, hLAT1 could respond to metabolic changes via the interaction with ATP and increasing the distribution of essential amino acids to cells under conditions of energy sufficiency. This may have a relevant role also in the metabolic rewire typical of cancer cells^52^. Furthermore, the accessible hydrophilic pocket identified in this work opens perspectives for the design of potential interactors able to target specifically hLAT1 contributing to the design of drugs for its silencing.

## Methods

### Materials

*E. coli* Rosetta(DE3)pLysS cells were from Novagen (Rome, Italy); His Trap HP and PD10 columns were from GE Healthcare; l-[^3^H]histidine was from American Radiolabeled Chemicals (ARC Inc., USA); C_12_E_8_, Amberlite XAD-4, egg yolk phospholipids (3-sn-phosphatidylcholine from egg yolk), cholesterol, Sephadex G-75, imidazole, l-histidine and all the other reagents were from Merck KGaA (Germany).

### Extraction of hLAT1 from SiHa cells

SiHa cells were maintained in Dulbecco's Modified Eagle Medium as described in^3^. LAT1 was extracted from SiHa pellets, after solubilization with RIPA buffer and incubation for 30 min on ice. Proteins were quantified, after centrifugation (12,000*g*, 15 min, 4 °C), using the colorimetric method Lowry-Folin^3^.

### Construction and over-expression of recombinant hLAT1 proteins

hLAT1 has been mutated using PCR overlap extension method as described in^53,54^ by using primers reported in Table 1. hLAT1 WT and K204Q mutant have been over-expressed in *E. coli* Rosetta(DE3)pLysS as described in^55^.

**Table 1:** Sequences of primers used for mutagenesis.

LAT1 K204Q Forward	TTTGCGGCGGCGCAGCTGCTGGC
LAT1 K204Q Reverse	GCCAGCAGCTGCGCCGCCGCAAA

### Protein purification

hLAT1 WT and K204Q mutant were over-expressed in *E.coli* and purified as previously described^55^. ÄKTA start FPLC equipment was used for purification. The supernatant from solubilized cell lysate was loaded on a His Trap HP column (5 mL Ni Sepharose) preequilibrated with a buffer containing 20 mM Tris HCl pH 8.0, 10% glycerol, 200 mM NaCl, and 0.1% sarkosyl (10 mL). The column was washed with a washing buffer containing 20 mM Tris HCl pH 8.0, 10% glycerol, 200 mM NaCl, 0.1% DDM, and 3 mM DTE (10 mL). The protein was eluted by the washing buffer added with 400 mM imidazole (15 mL). Desalt of 2.8 mL of the purified protein was then performed using a PD-10 column. The desalting buffer contained 20 mM Tris HCl pH 8.0, 10% glycerol, 0.1% DDM, and 10 mM DTE.

### Liposome preparation

7.5 mg of cholesterol were added to 100 mg of egg yolk phospholipids and solubilized with 1 mL chloroform. After short incubation under rotatory stirring (1200 rpm, 30 °C, 5 min) open tube is dried O.N. at room temperature^56^. The lipid film was resuspended in 1 mL water (10% final concentration) and sonicated to form unilamellar liposomes as previously described^57^.

### Reconstitution of the hLAT1 transporter into proteoliposomes

The desalted proteins, hLAT1 WT and mutant K204Q, were reconstituted by removing the detergent from mixed micelles containing detergent, protein, and phospholipids by incubation with Amberlite XAD-4 in a batch-wise procedure, as previously described^21^. In brief: the mixture for reconstitution was composed of 4 µg purified protein in desalting buffer (150 µL), 100 µL of 10% C_12_E_8_, 100 µL of sonicated liposomes prepared as described in the above paragraph, 10 mM histidine, 10 mM DTE, and 20 mM HepesTris pH 7.0 (except where differently indicated) in a final volume of 700 µL. Amberlite XAD-4 (0.5 g) was added to this mixture and incubated for 90 min under rotatory stirring (1200 rpm) at 23 °C using a previously pointed out procedure^21^. This methodology allows inserting the structurally asymmetric protein, with an homogeneous orientation (right-side-out) into the membrane^19^.

### Transport measurements

Transport was assayed as previously described^21^. In brief: proteoliposomes (600 µL) were passed through a Sephadex G-75 column (0.7 cm diameter × 15 cm height) equilibrated with a buffer containing 20 mM Hepes Tris pH 7.0 and 10 mM sucrose. Eluted proteoliposomes were divided into aliquots of 100 µL for transport assay. [^3^H]-histidine (5 µM) was added to the proteoliposome samples for starting the transport and 15 µM Mercury(II) Chloride (HgCl_2_) was added to stop the transport according to the stop inhibitor method^21^. To remove the external (not taken up) radioactivity, 100 µL of each sample were passed through a Sephadex G-75 column (0.6 cm diameter × 8 cm height). Samples were eluted with 1 mL 50 mM NaCl in 4 mL of scintillation mixture for radioactivity counting. Calculated specific activity was expressed as nmol/mg at a given time or as nmol/mg/min in the case of transport rate measurement. Kinetic parameters were derived from data fitting in Michaelis–Menten equation using Grafit 5.0.13 software (Erithacus Software, West Sussex, UK).

### Computational analysis

#### Protein preparation

The three-dimensional coordinates of LAT1 (PDB ID: 6IRT)^15^ were downloaded, refined, and prepared within Maestro v11.3^58^ using Schrödinger Protein Preparation Wizard tool^59,60^ which consists of three essential steps: addition of hydrogens, optimization of hydrogen bonds by flipping amino side chains, correction of charges, and minimization of the protein complex. Default parameters were used. Chain A, corresponding to CD98 and all ligands in 6IRT were removed.

#### SiteMap

The hydrophobic and hydrophilic regions on the protein surface were investigated using SiteMap^29,30,61^. The prepared chain B of 6IRT was submitted to SiteMap using default parameters and ranked. 10 sites were identified with a minimum of 15 site points after generating a fine grid (i.e. corresponding of a grid spacing of 0.35 Å) and enabling the detection of shallow binding sites on the surface.

#### Preparation of ligands

The ligands cholesterol and ATP were downloaded from PubChem in sdf format. Subsequently, they were prepared using LigPrep^62^ by optimizing geometries of the ligands and assigning them appropriate protonation states^63^. Epik with default parameters was used^64^.

#### Receptor grid generation

Receptor grids were generated keeping the default parameters of van der Waals scaling factor 1.00 and charge cutoff 0.25 subjected to OPLS3 force field. A cubic box of specific dimensions (30 × 30 × 30 Å) centered around selected residues (L53, V56, V60, F200, K204, A207, L210, I211, I280, I284, L291) was generated for the protein.

#### Induced fit docking (IFD) extra precision (XP)

Docking analysis were performed using Induced Fit XP Docking^65^ protocol in Maestro^66,67^ . At first, a Glide docking was carried out^68^. On the basis of B-factor, side chains were trimmed and a van der Waals scaling factor of 0.50 was used for both receptor and ligand. The number of poses generated was set to 20. All residues within 5.0 Å of ligand poses were refined and side chains optimized using a Prime structure prediction. This procedure allows the binding site residues to better accommodate the various ligand poses, resulting in more optimized protein–ligand interactions. Finally, structures within 30.0 kcal/mol of the best structure, and within the top 20 structures overall were redocked using Glide XP. The ligand was docked into the induced-fit receptor structure and the results yielded an IFD score for each output pose.

#### Autodock Vina

Autodock Vina v1.1.2 was used to identifying ATP binding sites in LAT1 transporter in the presence of Cholesterol (pose with docking score − 5.54 of Cholesterol, from Maestro)^32^ using a blind docking procedure. The grid box, which covered the whole protein, has a size of 70 × 102 × 74 Å (x, y, and z) with spacing 1.0. After ligand preparation (see above), the ATP molecule was docked into refined LAT1. The best conformation space of the ligand was searched employing the Lamarckian Genetic Algorithm. Default parameters were used and 20 different conformers were generated for the ATP molecule.

#### Binding energy calculation

Molecular Mechanics Generalized Born Surface Area (MMGBSA) solvation^69^ were used to calculate the binding energies for the best poses of LAT1-cholesterol and LAT1-cholesterol-ATP complexes derived from Induced fit docking XP (i.e. docking score). Default parameters with VSGB 2.051 energy model^70^ and OPLS3e force field were applied.

#### Visualization of docking results

Molecular graphics and visualization of docking results were performed with the UCSF Chimera v.1.7 software (Resource for Biocomputing, Visualization, and Informatics, University of California, San Francisco, CA, USA)^71^.

#### Alignment

The amino acid sequence of SLC7 members and other orthologues were downloaded from UniProt and aligned through the Clustal Omega^72^.

#### Homology modeling

We used a previously published homology model of LAT1 described in^73,74^. Briefly, the model was built using MODELLER using the structure of the arginine/agmatine transporter AdiC from *E. coli* in the outward open conformation (PDB ID: 5J4N) as a template^75^.

### Statistical analysis

Results were analyzed by nonparametric Student’s t-test as described in figure legends.

## Supplementary information

Supplementary Figures.
