# Supplementary material for: ATP modulates SLC7A5 (LAT1) synergistically with cholesterol
Source: Sci Rep. 2020 Oct 7;10:16738. doi: 10.1038/s41598-020-73757-y (PMC7541457; doi:10.1038/s41598-020-73757-y)
Supplement: Supplementary file 1 — Supplementary Figures. [file 41598_2020_73757_MOESM1_ESM.docx]

**ATP modulates SLC7A5 (LAT1) synergistically with Cholesterol.**

Jessica Cosco^1a^, Mariafrancesca Scalise^1a^, Claire Colas^2^, Michele Galluccio^1^, Riccardo Martini^2^, Filomena Rovella^1^, Tiziano Mazza^1^, Gerhard F. Ecker^2^, Cesare Indiveri^1*^

^a^ These authors contributed equally to this work

^1^Department of DiBEST (Biologia, Ecologia, Scienze della Terra) Unit of Biochemistry and Molecular Biotechnology, via Bucci 4C, University of Calabria, 87036 Arcavacata di Rende, Italy.

^2^University of Vienna, Department of Pharmaceutical Chemistry, Althanstrasse 14, 1090 Wien, Austria

* Corresponding author:

Tel:+39-0984-492939. Fax: +39-0984-492911. E-mail: cesare.indiveri@unical.it.

**Supplementary figures**

**
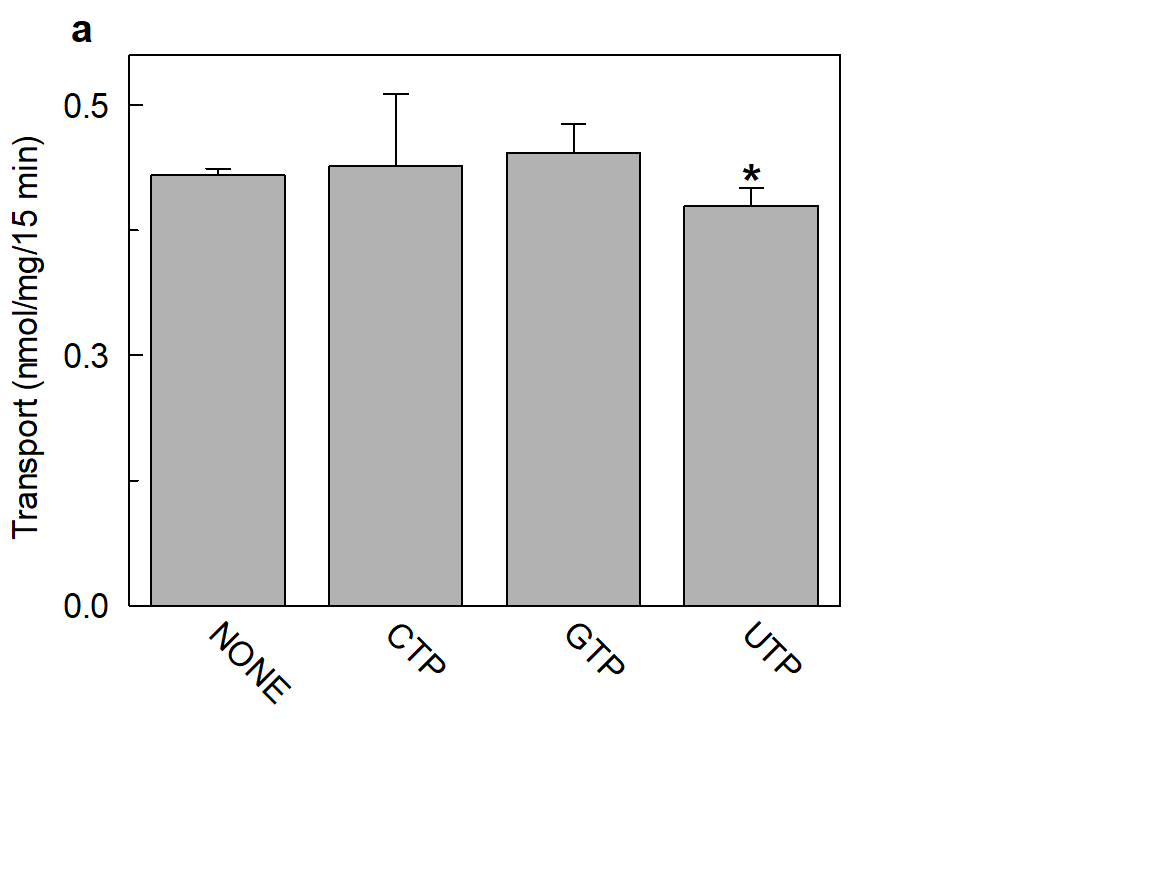
**

**
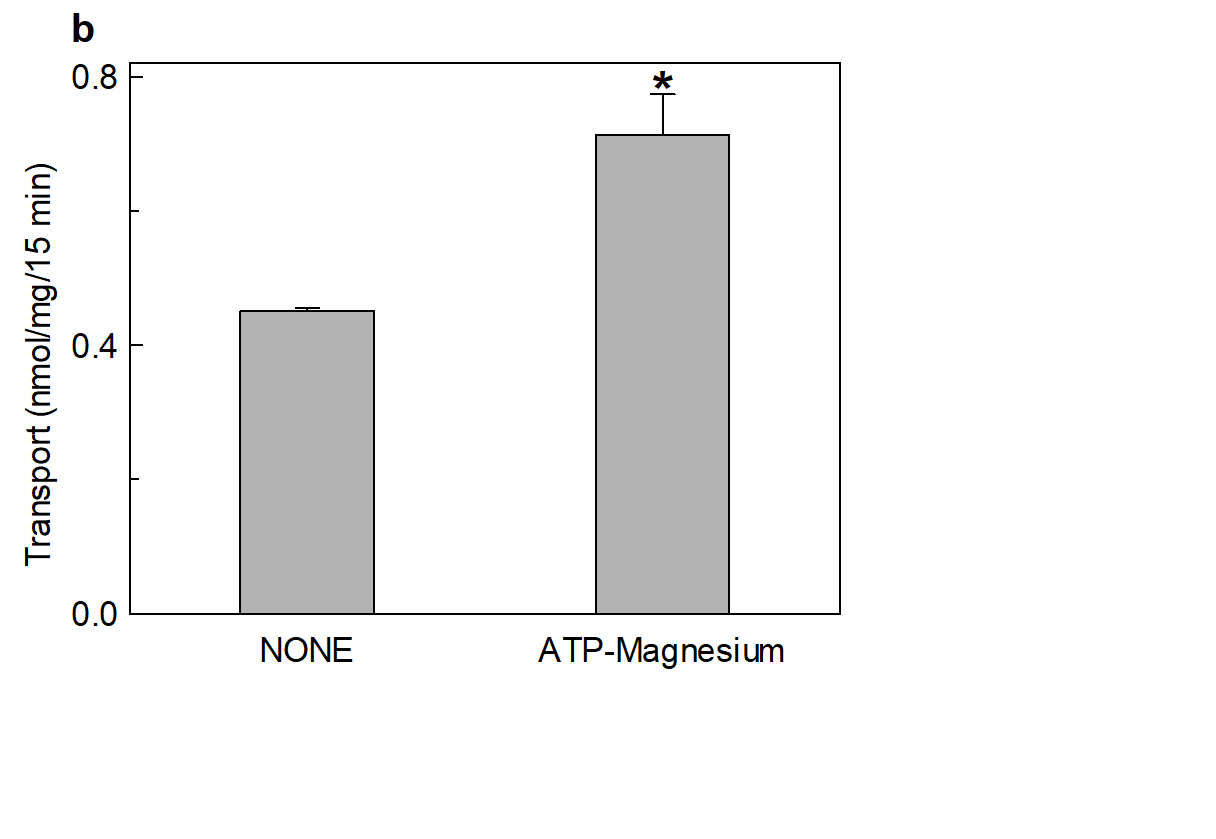
**

**Supplementary Figure 1**. Effect of nucleotides on the transport activity of hLAT1. The purified protein was reconstituted in proteoliposomes prepared with 75 μg cholesterol/mg phospholipids as described in Methods. Transport was started adding 5 μM [^3^H]-histidine to proteoliposomes containing 10 mM histidine. Transport was measured in 15 min according to the stop inhibitor method. Transport rate was expressed as nmol/mg in 15 min. In (**a**), the effect of nucleotides on the transport activity of hLAT1. Intraliposomal compartment included 0.3 mM of the indicated nucleotides, buffered with 20 mM HepesTris pH 7.0 In (**b**), the dependence of hLAT1 transport activity on intraliposomal ATP-Magnesium. The intraliposomal compartment included 4 mM ATP-Magnesium, buffered with 20 mM HepesTris pH 7.0. In (**a**) and (**b**), results are means ± SD of at least three independent experiments. (*) Significantly different from the control (no addition in the intraliposomal compartment, none) as estimated by the Student's t-test (p < 0.05).

**
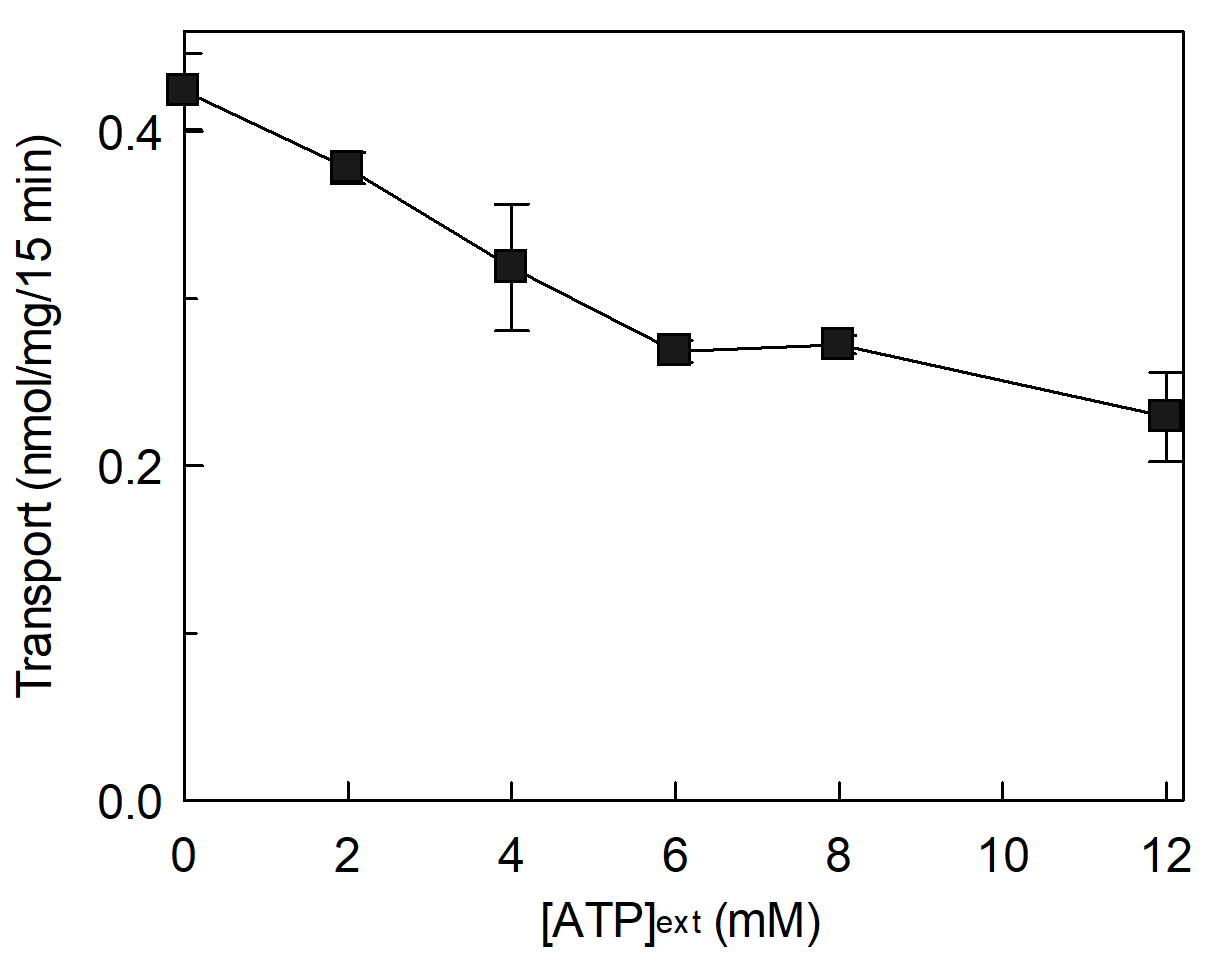
**

**Supplementary Figure 2.** Effect of extraliposomal ATP on the transport activity of hLAT1. The purified protein was reconstituted in proteoliposomes prepared with 75 μg cholesterol/mg phospholipids as described in Methods. Transport was started adding 5 μM [^3^H]-histidine, together with the indicated concentrations of ATP, to proteoliposomes containing 10 mM histidine and 4 mM ATP buffered with 20 mM HepesTris pH 7.0. The transport was measured in 15 min according to the stop inhibitor method. Transport rate was expressed as nmol/mg in 15 min. Results are means ± SD of at least three experiments.

**
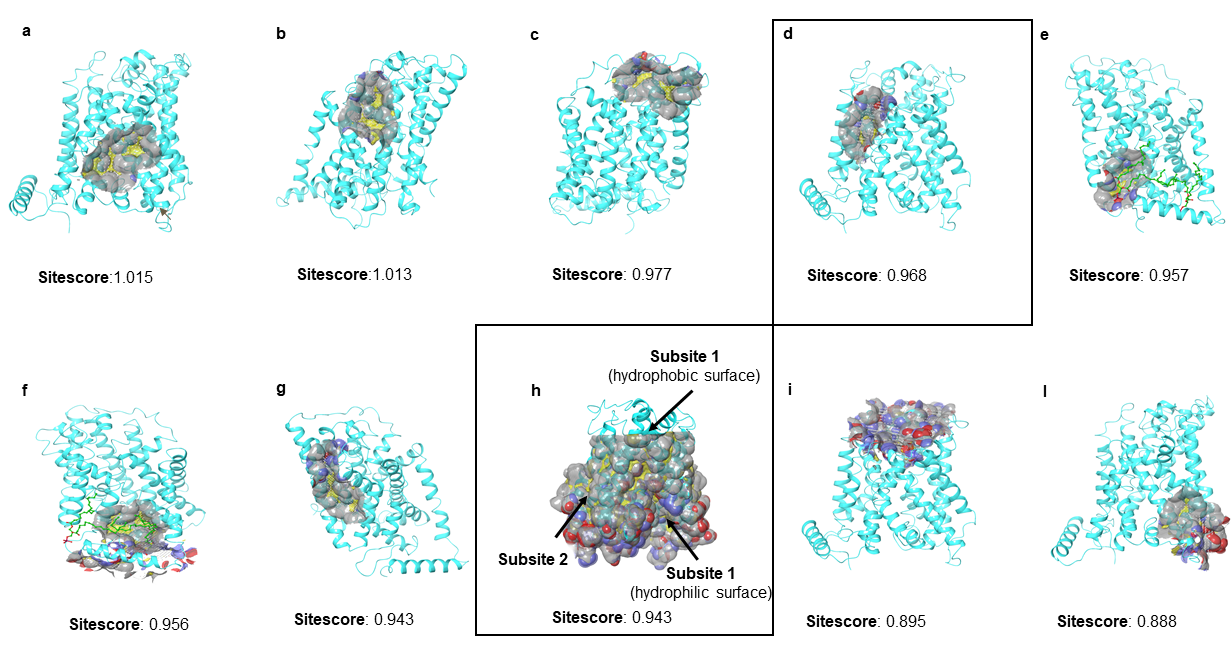
**

**Supplementary Figure 3**. Identification of hydrophobic and hydrophilic regions on the surface of LAT1. The crystal structure of hLAT1 in the inward open conformation (PDB ID: 6IRT, chain B) was represented as ribbon (sky blue) using Schrödinger-Maestro v11.3 ^58^. The analysis was performed using SiteMap ^61^, as described in Methods. Ten sites were reported and represented as surface on hLAT1 (**a, b, c, d, e, f, g, h, i, l**). In yellow, the hydrophobic sites; in red and blue the hydrophilic sites (hydrogen-bond acceptor in red and hydrogen-bond donor in blue). Sites **d** and **h** are highlighted with a black frame; subsites 1 and 2 are indicated in site **h**.


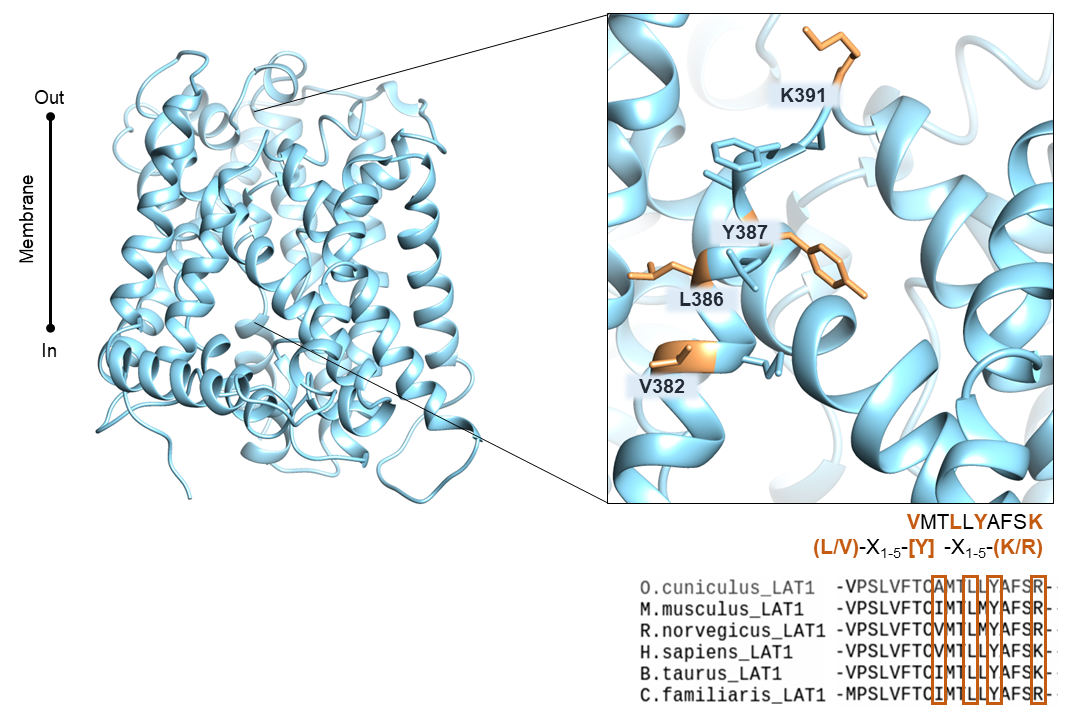


**Supplementary Figure 4**. Analysis of the potential cholesterol binding site **d**. The crystal structure of hLAT1 in inward open conformation (PDB ID: 6IRT, chain B) was represented as ribbon (sky blue) using Chimera v.1.7 software ([http://www.cgl.ucsf.edu/chimera](http://www.cgl.ucsf.edu/chimera/)). The membrane and intracellular/extracellular environment are indicated. In the zoom, identification of a CRAC motif (in orange) on TM 9 of hLAT1. Below, the alignment of hLAT1 with the other members of SLC7 family using Clustal Omega (https://www.ebi.ac.uk/Tools/msa/clustalo), as described in Methods.


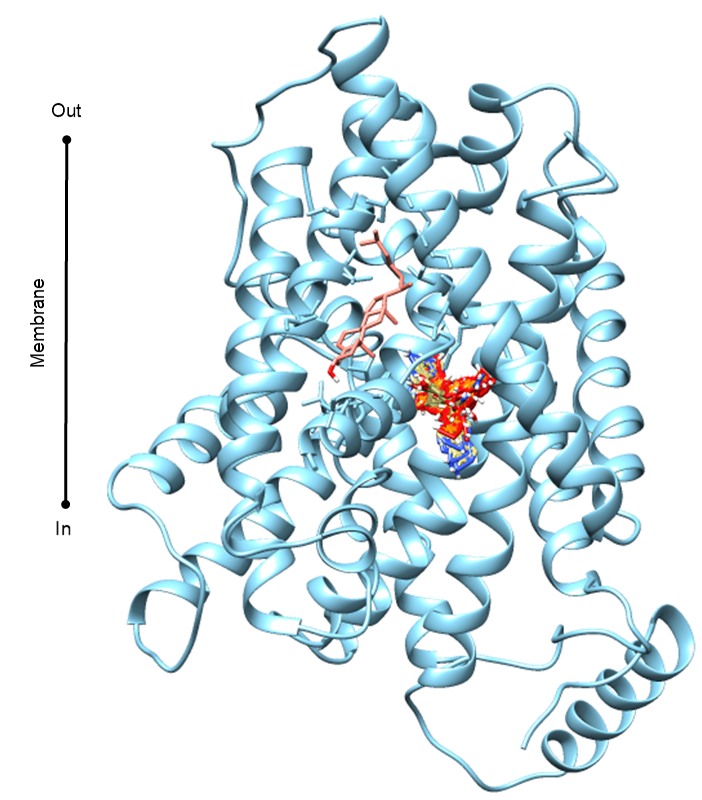


**Supplementary figure 5.** ATP docking results from AutoDock Vina. The crystal structure of hLAT1 in inward open conformation (PDB ID: 6IRT, chain B) was represented as ribbon (sky blue) using Chimera v.1.7 software ([http://www.cgl.ucsf.edu/chimera](http://www.cgl.ucsf.edu/chimera/)). Docking analysis was performed using AutoDock Vina v.1.1.2 ^32^ as described in Methods. The cluster of ATP poses was found close to cholesterol in subsite 1. The membrane and intracellular/extracellular environment are indicated.

**
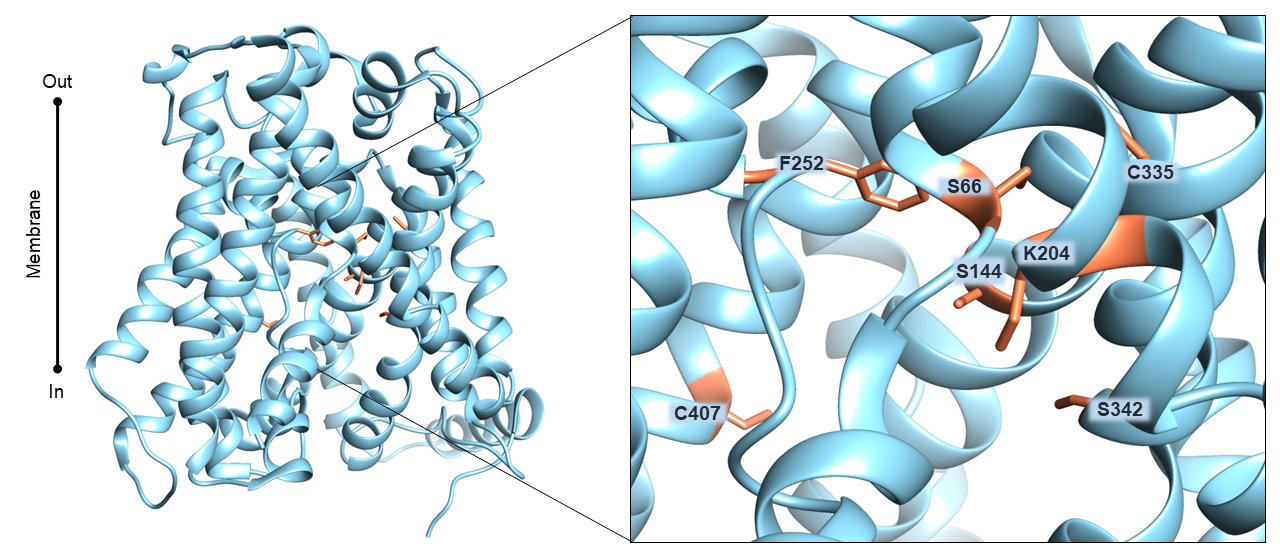
**

**Supplementary Figure 6.** Substrate binding site. The crystal structure of hLAT1 in inward open conformation (PDB ID: 6IRT, chain B) was represented as ribbon (sky blue) using Chimera v.1.7 software ([http://www.cgl.ucsf.edu/chimera](http://www.cgl.ucsf.edu/chimera/)). Docking analysis was performed using Induced Fit docking from Schrödinger-Maestro v11.3 ^65^ as described in Methods. On the right panel, a close-up view of the substrate binding site is shown. Residues belonging to the active site are represented as stick (coral) and labeled. The membrane and intracellular/extracellular environment are indicated.


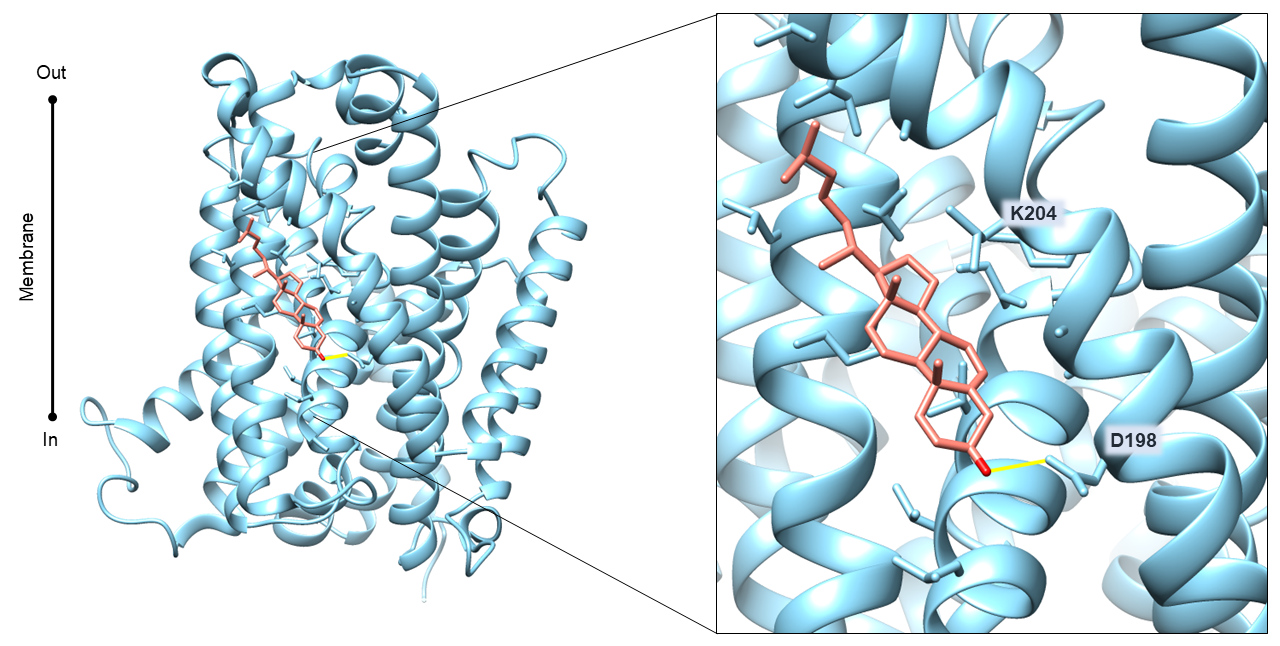


**Supplementary figure 7.** Docking of cholesterol on hLAT1 in the outward open conformation. The homology model of hLAT1 was represented as ribbon (sky blue) using Chimera v.1.7 software ([http://www.cgl.ucsf.edu/chimera](http://www.cgl.ucsf.edu/chimera/)). The homology model was built using the crystal structure of the prokaryotic homolog AdiC as template (PDB ID: 5J4N). Cholesterol docking in site 1 was performed using Induced Fit docking from Schrödinger-Maestro v11.3 ^65^ as described in Methods. The docking score is -6.021. The membrane and intracellular/extracellular environment are indicated.
